# Arthroplasty in Thumb (CMC Joint) Osteoarthritis: Avoiding Trapeziectomy

**DOI:** 10.1055/s-0045-1812997

**Published:** 2025-12-15

**Authors:** Marcio Aurelio Aita, Giulia Cordeiro Aita, Cleyton Rocha, Sullivan Savaris, Mauricio Leite, Samuel Pajares Cabanilla

**Affiliations:** 1 Division of Orthopedics, Surgery Department, Faculdade de Medicina do ABC, Santo André, SP, Brazil; 2 Medicine Program, Health School, Universidade Municipal de São Caetano do Sul (USCS), São Caetano do Sul, SP, Brazil; 3 Department of Orthopedics, Ortoclin, Camboriú, SC, Brazil; 4 Division of Orthopedics, SOS Mão, Recife, PE, Brazil; 5 Policlínica Gipuzkoa, San Sebastián, Spain

**Keywords:** arthroplasty, carpometacarpal joints, osteoarthritis, thumb, trapezium bone, articulações carpometacarpais, artroplastia, osteoartrite, polegar, trapézio

## Abstract

**Objective:**

To show the postoperative clinical results, including the patient-reported time to return to the activities of daily living (ADLs), the radiographic aspects, and the grip strength of total joint arthroplasty in patients with stage-III rizarthrosis.

**Methods:**

In the present single-center and prospective study, we analyzed total joint arthroplasty with an uncemented, metal-on-polyethylene prosthesis (Maia, Groupe Lepine). The inclusion criteria were patients with stage-III carpometacarpal (CMC) joint osteoarthritis, aged > 60 years, initially treated through non-surgical procedures. The exclusion criteria were patients presenting mental illness, alcohol abuse, rheumatological arthritis.

**Results:**

During the study period (from January 2018–October 2023), 34 patients met the patient selection criteria. Three eligible patients received a different implant, and two were simultaneously submitted to surgery in another joint (metacarpophalangeal joint with Z-deformity); these subjects were not included in the study. After 25.1 months of the surgery, grip strength was of 87.75% regarding the opposite side, the scores on the Visual Analogue Scale (VAS) and on the short form of the Disabilities of the Arm, Shoulder, and Hand (QuickDASH) questionnaire were of 1, and the range of motion was of 81% compared to the unaffected side. On the X-ray examination, initial metacarpal subsidence was observed in 100% of the patients. Complications were observed in 2 (6.9%) patients

**Conclusion:**

The choice of total joint arthroplasty to treat active patients aged > 60 years with stage-III CMC joint arthritis preserves independence to perform ADLs and improves quality of life in the first 12 months of the procedure.

## Resumo

**Objetivo:**

Mostrar os resultados clínicos pós-operatórios, incluindo o tempo de retorno às atividades da vida diária (AVDs) relatado pelo paciente, os aspectos radiográficos e a força de preensão, da artroplastia total em pacientes com rizartrose em estágio III.

**Métodos:**

Neste estudo prospectivo e unicêntrico, analisou-se a artroplastia total com prótese não cimentada de metal-polietileno (Maia, Groupe Lepine). Os critérios de inclusão foram pacientes com osteoartrite da articulação carpometacarpal (CMC) em estágio III, idade acima de 60 anos, tratados inicialmente com procedimentos não cirúrgicos. Os critérios de exclusão foram pacientes com doenças mentais, abuso de álcool e artrite reumatológica.

**Resultados:**

Durante o estudo (de janeiro de 2018 a outubro de 2023), 34 pacientes atenderam aos critérios de seleção. Três pacientes elegíveis receberam outro implante, e dois foram simultaneamente submetidos a cirurgia em outra articulação (metacarpofalangiana com deformidade em Z); estes indivíduos não foram incluídos no estudo. Após 25,1 meses da cirurgia, a força de preensão era de 87,75% em relação ao lado oposto, as pontuações na Escala Visual Analógica (EVA) e na versão curta do questionário de Incapacidade do Braço, Ombro e Mão (Quick Disabilities of the Arm, Shoulder and Hand, QuickDASH) eram de 1, e a amplitude de movimento era de 81% em comparação ao lado não acometido. No exame radiográfico, observou-se subsidência inicial do metacarpo em 100% dos pacientes. Dois pacientes (6,9%) apresentaram complicações.

**Conclusão:**

A escolha da artroplastia total para o tratamento de pacientes ativos com mais de 60 anos e artrite da articulação CMC em estágio III preserva a independência para a realização de AVDs e melhora a qualidade de vida nos primeiros 12 meses após o procedimento.

## Introduction

The prevalence of thumb basal joint arthritis increases with age and affects the ability to perform daily life activities (DLAs). 1 The number of patients seeking treatment for this condition is likely to increase. 2 However, there is no consensus regarding the prevention of total trapeziectomy in stage-III arthritis, according to the Eaton classification III.

There is doubt regarding the increase in complications and costs due to the complexity of the surgery. 2 Total joint arthroplasty (TJA) in trapeziometacarpal joint osteoarthritis can be a safe procedure in patients older than 60 years of age and in stage III after failure of the first non-surgical treatment. 3 4 5 6

In these cases, TJA is convenient; it does not hinder the performance of other techniques in case of failure, and it is a reproducible method that avoids stiffness, preserves the length of the thumb, increases the initial range of motion (RoM), enables a faster recovery, and it is an alternative to total trapeziectomy. 7

The objective of the present study was to present the postoperative clinical outcomes of ATJ, 2 including patient-reported time to return to DLAs, radiographic features and hand grip strength, in patients with stage-III rhizarthrosis.

### Increased Cost

The need for an implant (Maia prosthesis, Groupe Lepine) increases the cost of the treatment and makes health insurers reluctant to provide coverage to the patients, despite the growing evidence of its benefits.

## Methods

The current research followed the ethical standards and was approved by the institutional Ethics Committee on Human Experimentation. An informed consent form was provided to all research participants, who read and signed it according to their will.

In the present single-center prospective study, we analyzed TJA with an uncemented metal-on-polyethylene prosthesis (Maia, Groupe Lepine). The institutional Ethics in Research committee approved the free and informed consent form, which the patients signed before being included in the study. The inclusion criteria were patients with stage-III carpometacarpal (CMC) joint osteoarthritis, aged > 60 years, initially treated through non-surgical procedures. The exclusion criteria were patients presenting mental illness, alcohol abuse, and rheumatological arthritis.

During the study period (from January 2018 to December 2023), 34 patients met the selection criteria. Three eligible patients received a different implant, and two were simultaneously submitted to surgery in another joint (metacarpophalangeal joint with Z-deformity); these subjects were not included in the study.

### Post-hoc Analysis

The post-hoc analysis showed that 29 of the patients included did not show differences regarding age, sex, or CMC joint arthritis stage, with a statistical power of 85% according to a two-sided test and level of significance of 5%. 8 9 The sample was composed of 25 women and 4 men with a mean age of 64 (range: 60–74) years. Rhizarthrosis was classified according to the Eaton classification. 10 Follow up period was 25.1 [12-66] months.

### Surgical Technique

**Video 1** Thumb dynamic fluoroscopy: total joint arthroplasty enables the restoration of the length of the thumb and of tendon balance, the stabilization of the base of the thumb, and the increase in thumb abduction.


The goal of the treatment is to improve the balance between the mobility and stability of the CMC joint. Total joint arthroplasty must be perfectly positioned/fixated to enable osteointegration. 7

**Fig. 1:**
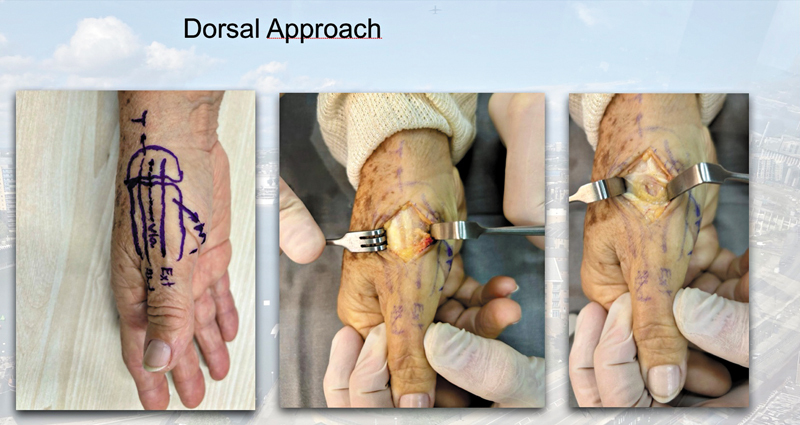
Dorsal thumb approach between the abductor pollicis longus and the short extensor tendons.

**Fig. 2:**
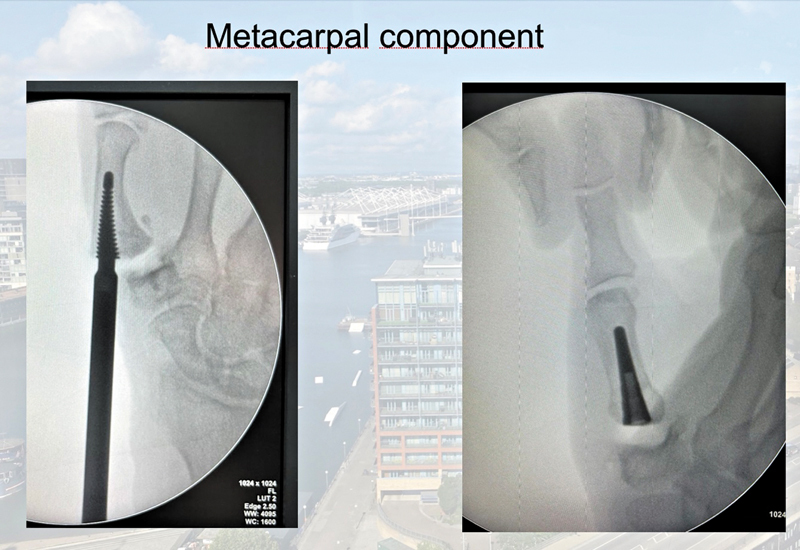
Metacarpal component.

**Fig. 3:**
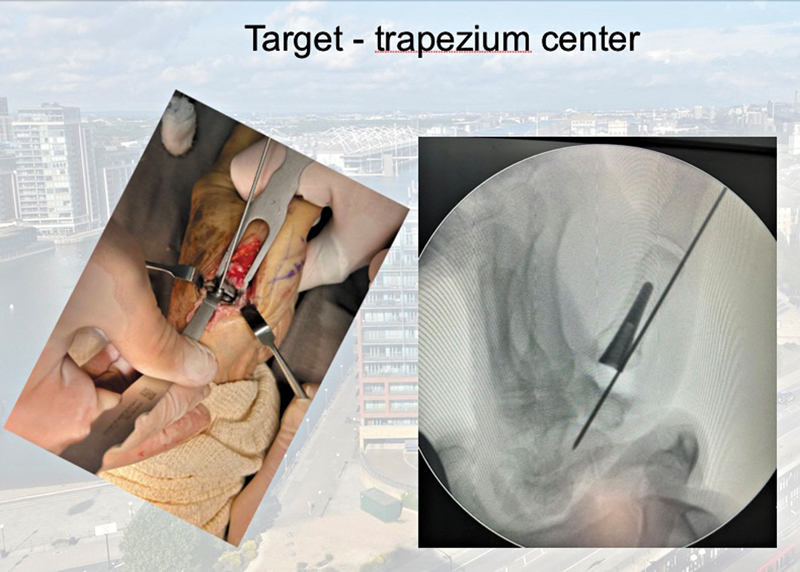
Trapezium component: center of motion of the carpometacarpal joint (procedure assisted by fluoroscopy).

The dorsal approach was chosen ( Fig. 1 ). The first step was to remove the joint surface of the base of the thumb, including the volar and medial beak osteophytes. Next, we prepared the medullary canal of the metacarpal with specific maneuver drills of increasing size until achieving press-fit stability and proper stem alignment along the metacarpal axis. The final implant was inserted, flush with the metacarpal base ( Fig. 2 ). Subsequently, we performed the cup placement in the trapezium taking care to avoid mechanical fixation of the cementless cup with the central subchondral bone and distal articular surface of the trapezium. The cup must be perfectly centered in the trapezium and in the center of motion of the CMC joint. To pass guide wire into trapezium center and the best direction that guide wire is 30° radially between the longitudinal axis of the diaphyses of the first and second metacarpals (coronal plane) and the anterior axis (sagittal plane), aided by fluoroscopy ( Figs. 3 4 ). Partial resection of the joint capsule was performed, with removal of free bodies and preservation of the palmar ligaments. A balance between soft tissues and implants is necessary to improve stabilization and avoid stiffness. The length of the thumb CMC joint can be assessed by comparing the length of the first and second rays before and after implanting the components. This can also be assessed by fluoroscopy, with analysis of the congruence of the first metacarpal arch on anteroposterior views with the thumb in 45° abduction, such as the “gothic arc” ( Fig. 5 ).

**Fig. 4:**
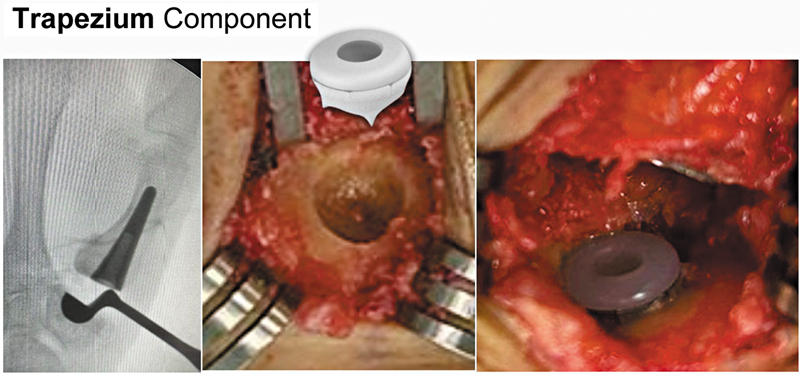
Trapezium component: the cup needs to be well centered and fixed (“press fit”) in the trapezium.

**Fig. 5:**
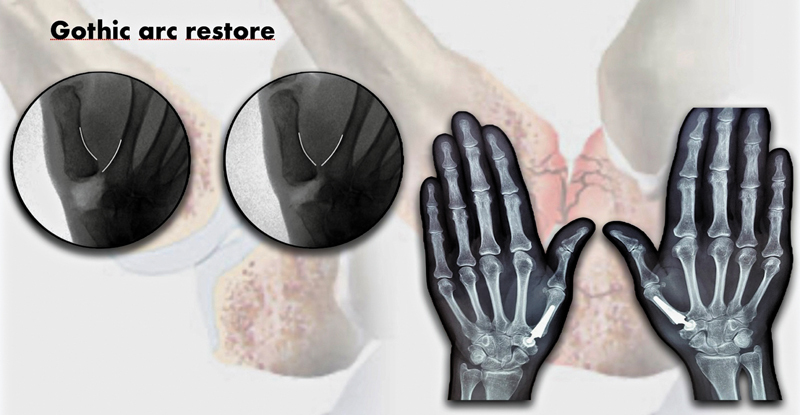
“Gothic arc”: congruency of the first and second metacarpal arches on anteroposterior view with the thumb at 45° of abduction.

Total joint arthroplasty allows the restoration of the length ( Video 1 ), tendon balance, stabilization of the CMC joint and increases the best range of motion of the thumb. 7

### Postoperative Care

A long thumb thumb splint was applied at the end of surgery and maintained for two weeks postoperatively. Skinsutures were removed so that the patient could actively move the thumb. A removable short thumb plaster cast was placed to be worn at nighttime and during DLAs. Pinching movement should be encouraged after 3 weeks, after which patients have no further restrictions.

All patients were assessed radiographic and clinically at 6 weeks, and again at 6 and 12 months after the procedure. 11 Range of motion (RoM) was evaluated with a single tool (goniometer). Grip strength was measured with a specific Jamar hand dynamometer (Sammons Preston, Bolingbrook, IL) and value obtained was expressed as a percentage of that presented on the opposite side. 12 13 These values were classified into 4 groups according to the grip strength quartiles to in relation to the time of return to DLAs. 14

The clinical outcomes were assessed using the Visual Analog Scale (VAS) for pain (range: 1–10, according to which 1 indicates no pain). Quality of life was determined by the Disabilities of the Arm, Shoulder and Hand (QuickDASH) questionnaire (range: 0–100, according to which 1 indicates the best result). 15 16 The rate of complications was assessed 6 weeks, and again at 6 and 12 months after the procedure.

### Radiographic Parameters

The main parameters were dorsal subluxation, implants aspect, and metacarpal subsidence (to maintain the trapezium-metacarpal joint space), which were evaluated at 6 weeks, and 6 and 12 months after the surgery.

### Statistical Methods

Table 1 shows the baseline demographics and the details of the injuries of the sample. The data were presented as mean or median according to the type of variable and its distribution. The objective and patient-reported outcomes are presented in Table 2 . The mean VAS and QuickDASH scores were of 1 point. The RoM was 81% compared with the nonaffected side. Radiographic examination to preserve the initial parameters (implants without dislocation and without failure) showed metacarpal subsidence in 100% of patients. Complications were observed in 2 (6.9%) patients. These included trapezium fracture during surgery, which was treated with K-wire fixation and bone consolidation. Another patient presented pain and decreased thumb abduction and was treated with rehabilitation exercises, but maintained VAS = 3.

**Table 1:** Demographics of the sample, side of the lesions, and associated diseases

Identification	Age (years)	Profession	Side	Sex	Associated diseases
1	69	Cook	Right	Female	—
2	63	Secretary	Right	Female	—
3	64	Designer	Right	Female	—
4	63	Designer	Left	Female	—
5	61	Secretary	Left	Female	—
6	60	Teacher	Left	Female	—
7	63	Teacher	Right	Female	—
8	60	Businesswoman	Right	Female	—
9	66	Accountant	Left	Female	—
10	62	Secretary	Left	Female	—
11	66	Nurse	Left	Female	—
12	60	Saleswoman	Right	Female	CTS
13	63	Dressmaker	Right	Female	—
14	70	Judge	Left	Female	—
15	64	Businesswoman	Left	Female	CTS
16	67	Maid	Right	Female	—
17	62	Maid	Right	Female	De Quervain tenosynovitis
18	63	Cook	Right	Female	—
19	67	Accountant	Right	Female	—
20	70	Judge	Right	Male	De Quervain tenosynovitis
21	64	Pastry chef	Right	Female	—
22	60	Driver	Right	Male	—
23	62	Seller	Right	Female	Ganglion
24	60	Businesswoman	Left	Female	—
25	70	Judge	Left	Male	CTS
26	64	Maid	Left	Female	De Quervain tenosynovitis
27	63	Designer	Left	Female	DIP arthritis
28	66	Maid	Right	Female	CTS
29	62	Businessman	Right	Male	—

**Abbreviations** : CTS, carpal tunnel syndrome; DIP, distal interphalangeal.

**Table 2:** Objective and patient-reported outcomes

Identification	Follow-up (months)	RoM (% opposite side) at 12 months	Quick DASH score	VAS score	Grip strength (% opposite side)	|Return to ADLs (months)	Complications
1	39	81	1	1	96	1	—
2	40	90	1	1	97	2	—
3	34	84	1	1	94	1	—
4	34	88	1	1	96	1	—
5	31	91	1	1	94	2	—
6	31	90	1	1	94	2	—
7	29	82	1	1	98	1	—
8	29	78	1	1	97	2	—
9	20	82	1	1	99	1	—
10	66	88	1	2	83	5	Trapezium fracture
11	76	91	1	1	91	1	—
12	36	80	1	1	96	1	—
13	14	78	1	1	92	1	—
14	12	74	1	1	94	2	—
15	17	79	1	1	96	1	—
16	16	82	1	1	98	1	—
17	18	83	1	1	87	2	—
18	19	91	1	1	98	2	—
19	14	90	1	2	96	1	—
20	14	90	1	1	96	1	—
21	14	82	5	3	87	6	—
22	24	88	1	1	96	1	—
23	21	73	1	1	91	1	—
24	20	89	1	2	83	1	—
25	12	70	11	2	82	5	Pain
26	12	64	1	1	81	1	—
27	12	63	1	1	96	2	—
28	12	66	1	1	92	1	—
29	12	62	1	1	97	1	—
**Mean**	**25.10**	**81**	**1**	**1**	**87.75**	**2**	

**Abbreviations** : ADL, activities of daily living; DASH, Disabilities of the Arm, Shoulder and Hand; RoM, range of motion; VAS, visual analog scale.

## Discussion

In recent years, several studies have considered the outcomes of multiple total trapeziectomy techniques with suspensionplasty to be successful. However, recent studies 1 2 11 have proven unsuccessful and suggested other procedures (which do not prevent other techniques from being performed in case of failure) to allow for faster recovery, with less pain and greater thumb strength, such as arthroscopy/button suture or total arthroplasty..

Grip strength is a valid and reliable method used to evaluate objective outcomes and is an independent predictor of of patients' inability to perform DLAs. Grip strength in the lower quartiles (first or second) increases the risk of inability to perform DLAs compared to strength in the higher quartiles (third or fourth). 13 14 In the present study, all patients showed values in the fourth quartile.

Bricout and Rezzouk 17 reported a failure rate of 7.7% in the implementation of the MAIA prosthesis in a series of 156 patients. Maeda et al. 11 showed a complications rate of 9.3%. We observed a lower incidence of complications (6.9%), and the clinical outcomes showed best RoM, shorter return time to DLAs, and a lower VAS than other studies. 2 6 7

A criticism of the reduction in pain, complication, and costs of traditional total trapeziectomy is clear, but nowadays, it is not only about reducing pain but also maintaining grip strength, range of motion and functional capacity to perform ADLs is to maintain trapezialmetacarpal length and reducing the dorsal subluxation of the thumb metacarpal 18 because increasing grip strength and it is appropriate to perform procedures that preserve the biomechanics of the thumb joint and, if it fails, we can perform another salvage procedure, such as preserving the trapezium by performing this prosthesis and, if this fails, a total trapeziectomy can be performed and thus we do not skip treatment steps. (“no burnt bridges” concept). Newton and Talwalkar, 2 Duerinckx and Verstreken, 7 and this study have demonstrated that TJA has certain advantages over other options, including stabilization and alignment of the CMC joint and preservation of the ability to perform DLAs and thumb length. Hustedt et al. 8 showed that the time of return to work was 4.5 months in patients who had undergone total trapeziectomy. In our study, 93.1% of those who underwent the procedure returned to work within 2 months.

Farkash et al. 19 reported a failure rate 11,32%, with these failed procedures in patients treated with partial arthroplasty of the CMC joint. The disadvantages of this method include costs, technical difficulty of the surgery, and the possible complication rate.

Currently, the concept “no burnt bridges” is accepted, and early definitive surgery in CMC joint arthritis, such as, total trapeziectomy, is controversial, because their exhausts surgical treatment options. This concept recommends the use of thumb arthroscopy or arthroplasty 20 21 whenever possible. This approach improves the patients' quality of life and reduces recovery time for DLAs and work-related activities. In addition, in case of failure, all surgical revision methods can be performed.

The present study is a prospective clinical trial, and all patients were operated on by a single hand surgeon, constituting a uniform group with complete follow-up. Nonetheless, some limitations need to be recognized. The present study is a case series and not a randomized clinical trial, the inclusion criterion was stage III according to the Eaton classification, and the sample size was small for QuickDASH, VAS, and grip strength analysis.

The increased cost is the main reason behind the difficulty in performing TJA. The method and implant for the treatment of thumb arthritis have evolved exponentially, generating excellent and are an option before comparative studies can consider them the gold standard and procedure of choice. 21

## Conclusion

The choice of TJA to treat active patients > 60 years old, with stage-III CMC joint arthritis, helps to preserve independence to perform DLAs and improve the patients' life quality in the first 12 months after the procedure.
